# Negation and Negative Concord in Georgian Sign Language

**DOI:** 10.3389/fpsyg.2022.734845

**Published:** 2022-07-14

**Authors:** Roland Pfau, Tamar Makharoblidze, Hedde Zeijlstra

**Affiliations:** ^1^Department of Linguistics, University of Amsterdam, Amsterdam, Netherlands; ^2^School of Arts and Sciences, Ilia State University, Tbilisi, Georgia; ^3^Seminar for English Philology, Georg-August-University, Göttingen, Germany

**Keywords:** negation, negative concord, Georgian Sign Language, modality, tense, sign language typology

## Abstract

Negation is a topic that has received considerable attention ever since the early days of sign language linguistics; also, it is one of the grammatical domains that has given the impetus for sign language typology. In this paper, we offer a typological and theoretical contribution to the study of sign language negation. As for the typological side, we add Georgian Sign Language (GESL) to the pool of languages investigated. Our description reveals that GESL displays a number of typologically unusual features: a considerable number of negative particles, including emphatic, prohibitive, and tense-specific particles; specialized negative modals; and a wide range of possibilities for Negative Concord (NC) involving two manual negative signs, including a unique tense-specific instance of NC. Most of the patterns we report—available negative particles, their clausal position, and NC possibilities—are clearly different from those attested in spoken Georgian. As for the theoretical contribution, we investigate how the highly complex GESL negation system compares to existing taxonomies of NC and Double Negation systems, and we conclude that GESL aligns with certain languages that have been classified as atypical NC languages.

## Introduction

Even after 60 years of linguistic study, many aspects of the grammars of natural sign languages still have either not been thoroughly investigated at all, or only for a small number of (mostly Western) sign languages. Clausal negation, however, is a domain of grammar that has been comparably well studied for a fair number of sign languages from different geographical regions, including some so-called village sign languages. Actually, next to interrogatives, negation is one of the domains of grammar that gave the impetus for sign language typology, a young and thriving research field (Zeshan, 2004a,b, 2006; De Vos and Pfau, 2015; Zeshan and Palfreyman, 2017). Notably, clausal negation is also a prominent domain of inquiry in spoken language typology (e.g., Payne, 1985; Dryer, 2005; Miestamo, 2005; Dahl, 2011). Efforts have been made to compare the realization of clausal negation across language modalities, that is, to investigate in how far patterns attested in sign languages (visual–spatial modality) fit, or do not fit, into typological classifications put forward on the basis of a large number of spoken languages (auditive-vocal modality). Despite the use of resources that appear to be modality-specific, such as non-manual markers (for example, brow, head, and torso movements; *cf.* Pfau and Quer, 2010), it has been suggested that typological classifications can be applied to sign languages (e.g., use of negative particles and affixes and French-style split negation (*ne … pas*); *cf.* Pfau, 2008, 2015; Gökgöz, 2021). However, this does not exclude the possibility that we also find patterns that are either specific to sign languages as a group (i.e., modality-specific patterns) or to a particular sign language.

In this paper, we add to the typological picture data from Georgian Sign Language (GESL), an as yet understudied sign language. On the one hand, we sketch how basic clausal negation is realized in this language, and we conclude that GESL can be classified as a sign language of the manual dominant type. On the other hand, we zoom in on the interaction of negation with other grammatical categories, namely, tense, aspect, and modality. It is the latter domain of inquiry that presents us with some typologically unique features—unique not only in comparison with other sign languages, but also in comparison with spoken languages. Throughout, we include in the presentation various types of Negative Concord that are attested in the language.

In the remainder of the introduction, we briefly introduce GESL and sketch some general characteristics of sign language negation. In “Negation in Spoken Georgian”, we describe how clausal negation is realized in spoken Georgian. This is important, as it will allow us to evaluate whether certain patterns that we identified in GESL are possibly the result of language contact. In “Methodology”, we explain our methodology. In “Word Order and Basic Negation in GESL”, we then turn to a description of word order facts and the realization of basic negation in GESL. The complex patterns of interaction of negation with tense, aspect, and modality, including various types of Negative Concord, are detailed in “On the Interaction of Negation With Tense, Aspect, and Modality”. In “Discussion”, we investigate how the highly complex GESL negation system compares to existing taxonomies of Negative Concord and Double Negation systems.

### Georgian Sign Language

GESL is the sign language used by Deaf and hard-of-hearing people in Georgia. At present, it is unknown how many people use GESL for communication in daily life, but it is estimated that at least 2,500 people use GESL on a regular basis. In the Georgian constitution, GESL is not mentioned as an official language of Georgia. However, in recent years, GESL has received more and more official recognition—also thanks to linguistic research on the language. It is, for instance, mentioned in various governmental documents of the State Language Department and of the Ministry of Education and Science. It is also the official language of instruction at the three deaf schools in Tbilisi, Kutaisi, and Batumi.

Before becoming independent in 1991, Georgia was part of the Soviet Union, and it is therefore not surprising that GESL has been influenced by Russian Sign Language, especially at the lexical level—similar to other sign languages in former parts of the Soviet Union. This influence notwithstanding, the available evidence suggests that GESL is an independent language, which has actually been gaining strength in recent years, emancipating itself from the Russian Sign Language influence—also thanks to activities of the local Deaf community.

To date, only a few linguistic studies on GESL are available. In 2012, an overview of the language, including sociolinguistic information and a sketch of its grammar, has been published (Makharoblidze, 2012), followed by the publication of a GESL-Georgian dictionary with 4,000 entries (Makharoblidze, 2015a; see http://gesl.iliauni.edu.ge/ for the online version). As for studies on aspects of GESL grammar, Makharoblidze (2015b) describes the use of a number of indirect object markers, Makharoblidze and Pfau (2018) address the interaction of negation with tense (which is also part of the present study), and Makharoblidze (2019) provides an overview of verbal morphology.

### Sign Language Negation

As mentioned before, the fact that negation is comparably well studied for sign languages—for individual sign languages as well as from an intra-modal comparative perspective—allows us to extract certain recurring typological patterns. We start by noting that all sign languages studied to date employ manual negative markers as well as non-manual markers, mostly a side-to-side headshake, in the realization of clausal negation. The way in which these two types of markers interact, however, has been shown to be subject to language-specific rules (Pfau and Quer, 2002; Zeshan, 2004a; Pfau, 2015, 2016).

First, in some sign languages, the use of a manual negative element is optional. In Sign Language of the Netherlands (*Nederlandse Gebarentaal*, NGT), for instance, the negative particle not may be used (1a,b), but clausal negation is more commonly realized by means of only a headshake, which simultaneously accompanies one or multiple manual signs (1c; Oomen and Pfau, 2017, p. 21, 23). In contrast, it is not possible to negate a clause only by means of not, i.e., without headshake. The corpus-based study by Oomen and Pfau reveals that the negator not mostly follows the verb (1a) but may also precede the VP (1b; Oomen and Pfau, 2017, p. 22). Furthermore, the headshake (“hs”) always accompanies not, and, in the absence of not, at least the verb, but it may also spread onto the object and/or clause-final pointing signs, like the repeated subject pronoun in (1c).^1^


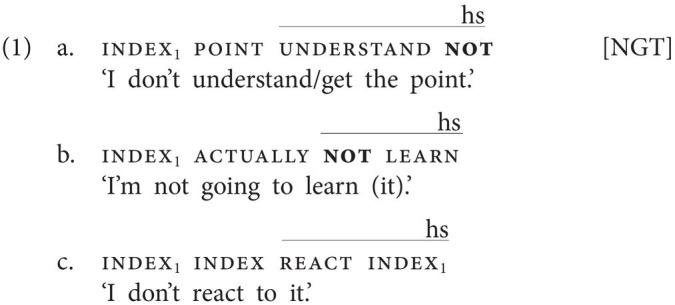


Sign languages like NGT, in which the use of a manual negative particle is optional and spreading of the headshake is possible, are referred to as “non-manual dominant” sign languages. Clearly, in sign languages of this type, the headshake carries negative force, as it can negate a proposition by itself, and it has therefore been suggested that examples like (1a, b) exemplify Negative Concord involving a manual and a non-manual negative marker (Pfau, 2016); see “Discussion” for further discussion.

This contrasts with “manual dominant” sign languages, in which the use of a manual negative sign is obligatory. Still, sign languages of this type also employ a headshake (or sometimes a backward head tilt), but this non-manual marker usually only accompanies the manual negator. The examples in (2) show that Italian Sign Language (LIS) belongs to this latter group. Crucially, (2b) is ungrammatical irrespective of the scope of the headshake (Geraci, 2006b: 221), showing that the headshake in LIS does not carry negative force.^2^


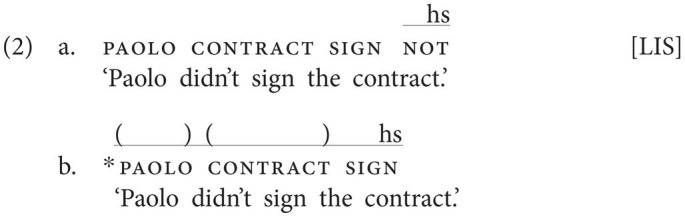


Based on the typological dichotomy and syntactic constraints imposed on the scope of the headshake, it has been claimed that in many sign languages, the headshake should be considered a grammaticalized gesture (Van Loon et al., 2014; Pfau, 2015). However, this need not be the case in all sign languages. For instance, based on corpus data, Johnston (2018) has recently argued that the headshake is not a grammatical marker of negation in Australian Sign Language, a manual dominant sign language: in this language, headshakes are observed in just over half of the manually negated clauses (in striking contrast to NGT), and their position and spreading behavior do not appear to be linguistically constrained.

Numerous sign languages have been reported to have at their disposal multiple negative particles, often expressing additional meanings, such as emphatic negatives, negative existentials, or particles with additional aspectual meaning. The NGT example in (3a) involves the negative completive marker not.yet (Coerts, 1992, p. 209), whose handshape and movement are different from that of the negative particle not. The use of an emphatic negative particle is illustrated by the Jordanian Sign Language (LIU) example in (3b); this particle differs from the basic negator not, which is also present in the example, in movement and accompanying facial expression [adapted from Hendriks (2008, p. 79); non-manuals not specified in original example; “//” indicates a prosodic break].


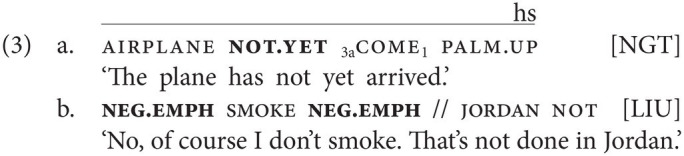


In addition, it is fairly common across sign languages to have special forms for negative modals, be it cliticized or suppletive forms (Shaffer, 2002; Zeshan, 2004a; Pfau and Quer, 2007). Such specialized manual negators will play a prominent role in our discussion of GESL negation in “Word Order and Basic Negation in GESL” and “Negative Modals”.

## Negation in Spoken Georgian

In this section, we sketch the realization of sentential negation in Georgian, the spoken language that GESL is in contact with, as we are also interested in possible language contact phenomena. Georgian has two basic negative particles: *ar(a)* “not” (which also functions as negative reply “no”) and *ver(a)*, which has a modal flavor and is often translated as “cannot,” although the modal meaning may at times be rather subtle. Both particles always immediately precede the lexical verb, as is shown in the examples in (4) and (5). In (5), we also illustrate the difference between the two particles. The version in (5b) is the neutral negative version; it simply implies that no letter writing has taken place, for instance, because the speaker did not want to. In principle, (5c) could receive the same translation, but it implies that there was an intention to write a letter and that specific reasons made it impossible (e.g., lack of time and no stationery available; prev = preverb and aor = aorist).


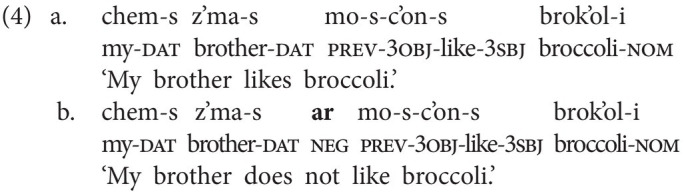



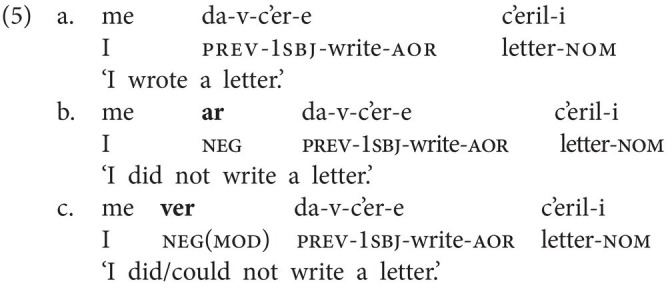


Word order in Georgian is fairly free. The above examples, and the ones to follow, display the common SVO order, but SOV is also attested (alongside other permutations). In both orders, the negative particles immediately precede the verb, that is, the standard orders in negated clauses are SNegVO and SONegV, respectively.

When neg-words or negative adverbials are used, Negative Concord (NC) is very common in Georgian, but it is not obligatory. This is illustrated for the neg-word *araperi* (“nothing”) in object position in (6) and for the negative adverbial *arasodes* (“never”) in (7; ver = marker of version). The (b) examples involve the negative particle *ar(a)*, but NC involving the particle *ver(a)* is also attested, as is shown in (6c) and (7c)—in this case, the neg-word adapts to the negative particle.^3^


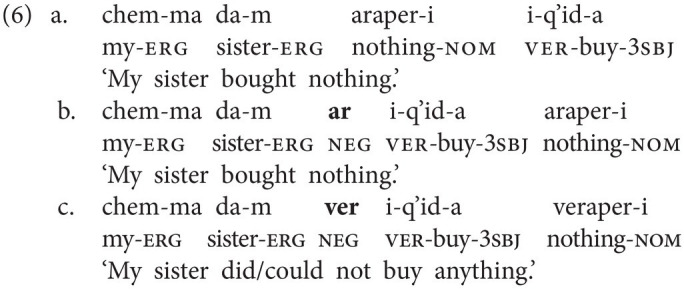



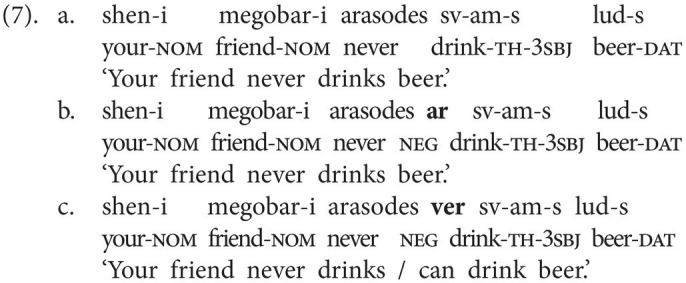


Besides the two particles mentioned above, Georgian has an additional negative particle, prohibitive *nu*, which can only be used in the imperative and which—just like the other particles—always immediately precedes the verb; *cf.* (8).


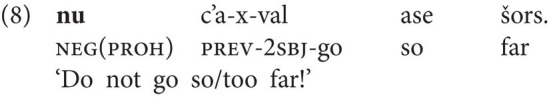


Further phenomena related to negation in spoken Georgian will be introduced in subsequent sections in order to scrutinize the degree in which spoken Georgian has possibly had an impact on the realization of negation in GESL. While it has long been demonstrated that natural sign languages generally do not copy the grammatical structure of the surrounding spoken language (e.g., word order and availability of certain grammatical categories), it is also clear that the spoken language may have an influence on the sign language (Plaza Pust, 2005; Adam, 2012)—and this is a possibility we want to explore for GESL.

## Methodology

### Spontaneous Data

Many of the patterns we describe in this paper were first observed in spontaneous narratives, about 5 h in total, produced by 15 native signers (age 24–65), which have been recorded for the purpose of studying the verbal morphology of GESL, as well as some sociolinguistic properties. All signers are from Tbilisi and are members of the Deaf Union of Georgia. They were asked to share personal experiences and/or anecdotes with a Deaf interlocutor.

The productions of the signers, which vary in length between 5 and 45 min, have been annotated in ELAN with the help of Deaf research assistants and GESL interpreters. All participants produced negative utterances. As for negation, the spontaneous data revealed (i) that GESL features two basic clause negators, which can appear in various positions within the clause; (ii) that, moreover, particles with additional semantics (e.g., emphatic) exist; (iii) that different types of Negative Concord are attested; and (iv) that GESL has specialized negative modals. Note that to date, only manual signs have been annotated, but all negative examples extracted from the data were checked for the presence of a headshake.

### Data Elicitation

Subsequently, the patterns concerning negation that we had extracted from the spontaneous data were supplemented by elicited data. Five GESL signers from Tbilisi (age 22–60), who had not been involved in the recording of spontaneous data, participated in an elicitation session, administered by a sign language interpreter, who is also a native signer. These five signers are born and raised in Deaf families and are actually either from the third or fourth Deaf generation within their family. They are also members of the Deaf Union of Georgia and are considered as the best GESL signers among the community members. Four of them teach GESL to other Deaf and hard-of-hearing people at the Deaf Union and/or at Deaf schools.

Data elicitation involved two different approaches. On the one hand, participants were shown negative clauses from the spontaneous data and were asked to repeat them. Each participant saw between 60 and 90 negative clauses, distributed over multiple sessions. If the participant changed the structure during repetition (e.g., different word order and different or additional particle), they were asked why they implemented the change; if the participant repeated verbatim, they were asked whether an alternative structure would be possible. On the other hand, the sign language interpreter presented to the participants affirmative clauses, which were modeled based on negative clauses extracted from the spontaneous data, and participants were asked to negate these clauses. However, some of the model clauses contained time adverbials which were not present in the original example. Each participant saw between 80 and 120 such (modified) affirmative clauses, again spread over various sessions.

The elicited data confirmed the patterns we had previously observed (e.g., basic negation strategy and Negative Concord), but also presented us with additional negation strategies (e.g., additional specific negative particles). In the first elicitation task, signers would, for instance, indicate that another particle could be used or that two manual negators could be combined. The second elicitation strategy confirmed the existence of negative modals and brought to light some further unexpected findings, such as the existence of a tense-specific negation strategy.

### Grammaticality Judgments

In a third step, we also obtained grammaticality judgments by the same five signers on pre-recorded sentences, produced by the before-mentioned GESL interpreter, which either mirrored the negation patterns found in the spontaneous and elicited data, or in one way or the other deviated from them. The deviations were implemented to test the (un)grammaticality of certain structures which had surfaced in the spontaneous data and during elicitation. This allowed us to further confirm these patterns and also to identify ungrammatical structures. Deviations involved, for example, a change in word order, the addition of a time adverbial, and/or the addition of another negative particle.

The same five signers who participated in the elicitation tasks also participated in the grammaticality judgment task. Each of them was presented with 120 examples. Judgment did not involve the use of a Likert-scale, but only absolute statements in the form of “acceptable / unacceptable / unclear.” Remarkably, judgments of the signers were almost unanimous (97% agreement), in particular regarding the combinatory possibilities of manual negators and the tense-specific strategy which had been identified during elicitation. One has to keep in mind, of course, that all signers participating in the grammaticality judgment task came from Tbilisi. It may well be the case that signers from other regions would offer different judgments for some of the examples.

## Word Order and Basic Negation in GESL

### Word Order in Affirmative Clauses

Similar to what we described for Georgian, word order is also free in GESL. Besides SVO and SOV orders, V-initial and O-initial orders are also attested—albeit less frequently—where the latter order arguably results from topicalization (though information structure has not yet been fully investigated for GESL). Napoli and Sutton-Spence (2014) demonstrate that across sign languages, it not at all uncommon to find both SVO and SOV within a single language, but that generally, the order is less constrained for verbs that allow spatial modification to indicate their arguments, i.e., so-called “agreeing” or “indicating” verbs. In a nutshell, in these verbs, the start point of the verb’s movement trajectory typically aligns with the locus in space associated with the subject, while the end point aligns with the locus associated with the object.^4^ GESL also distinguishes verbs that can be modified in this way (e.g., talk.to, answer, and give) and verbs that cannot be spatially modified (so-called “plain” verbs, e.g., like, understand, and help). Interestingly, however, in GESL, word order is free with all verbs, as is shown in (9) for the plain verb like and in (10) for the agreeing verb talk.to. Sentence adverbials commonly occupy a clause-initial position (10), but they may also appear clause-finally.


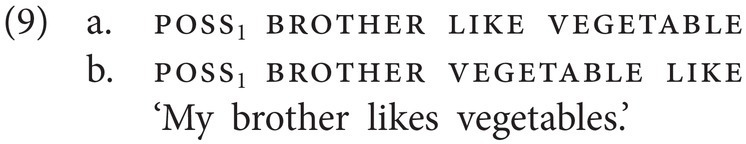



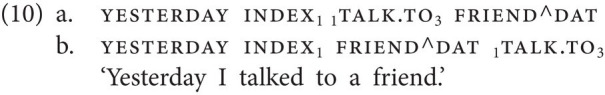


Note that GESL has a rich system of manual case markers that only combine with animate arguments and that may cliticize to the noun they accompany. We shall not discuss these markers in detail, as they are not relevant in the present context (see Makharoblidze, 2015b). Still, as some of the examples we present include such markers, and given that some informants judge at least some examples as marked or even ungrammatical when the case marker is omitted, they have to be mentioned. The dative marker in (10), for instance, involves a 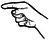-handshape, which cliticizes to the noun friend; cliticization is realized by a continuous movement contour from the noun to the case marker, such that the latter loses its syllabicity (cliticization is indicated by “^”).

### Basic Negation

**Figure 1:**
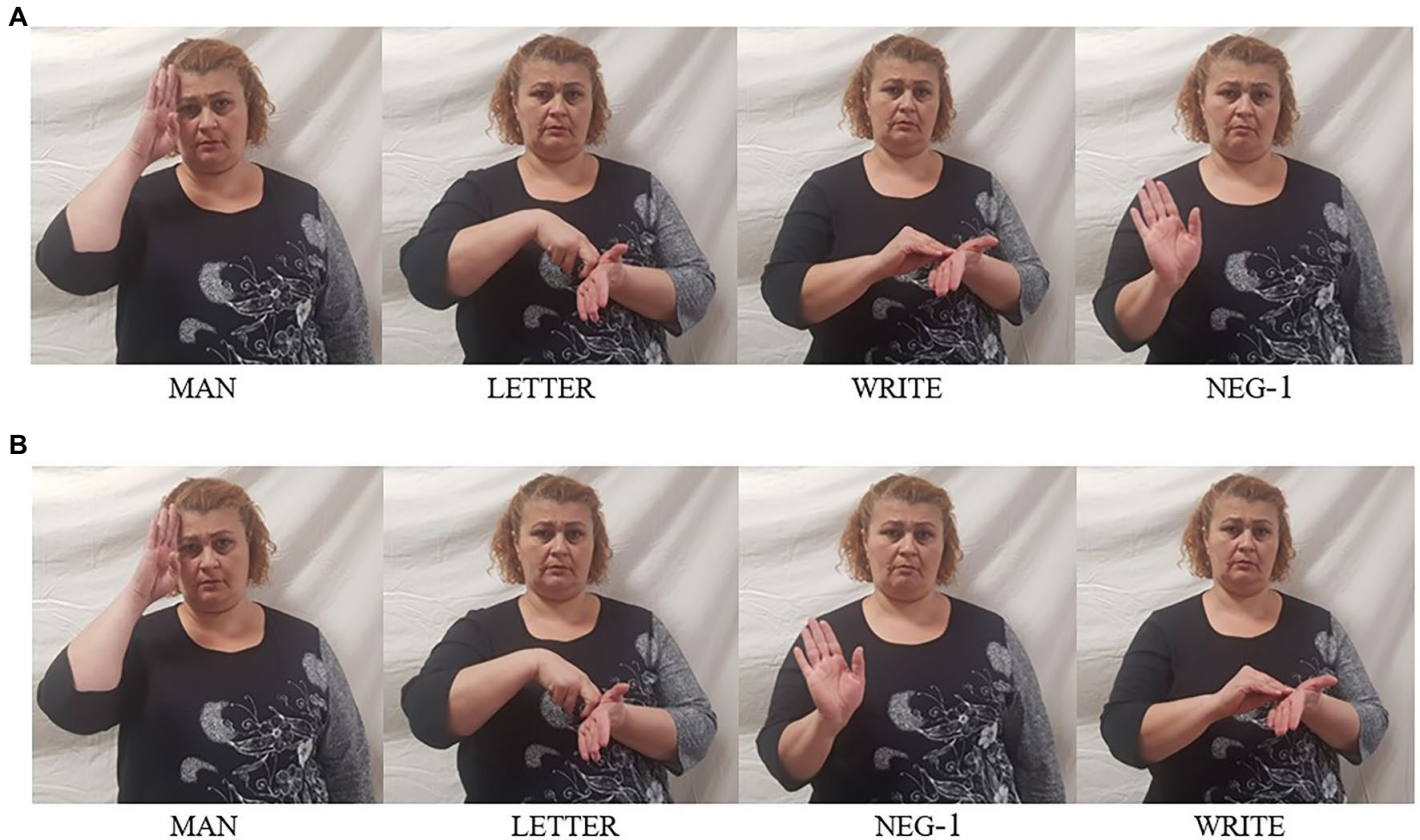
Negated transitive clause “I do/did not write a letter,” with **(A)** negative particle following the verb and **(B)** negative particle preceding the verb.

The basic clause negator in GESL, which we gloss as neg-1, is articulated with a flat hand (all fingers extended, palm facing forward), which executes a small repeated shaking movement resulting from rotation of the lower arm. This particle usually appears clause-finally, but it may also precede the verb, as is shown by the two examples in Figure 1, which express exactly the same meaning. Both examples display OV order, but given that VO order is also possible, other attested orders are SVONeg and SNegVO. Remember from the discussion in “Negation in Spoken Georgian” that of these four orders, spoken Georgian only allows those in which the negative particle immediately precedes the verb (i.e., SNegVO, as in (4b), and SONegV).

Such a variable position of the basic clause negator, without semantic impact, has also been described for other sign languages. For instance, in NGT, a sign language which allows for OV and VO order, the particle not also most commonly appears clause-finally, but in contrast to GESL, its alternative position is preceding the entire VP (Oomen and Pfau, 2017); the opposite pattern has been described for American Sign Language (ASL; Wood, 1999). It is not really clear what underlies this variability; while Oomen and Pfau assume that pre-VP placement results from Neg-movement, Wood argues that sentence-final placement of not is derived by VP-movement to a position preceding the negator.

Judgments by all of our informants indicate that GESL has to be classified as a manual dominant sign language. They unanimously agree that examples like those in (11) are ungrammatical—irrespective of word order and irrespective of the exact spreading domain of the headshake (which, in the below examples, is the VP). In other words, the headshake by itself does not contribute negative force, and therefore, a manual negator is required in the expression of clausal negation. Moreover, all the examples we extracted from the data include a headshake, and it appears (i) that the headshake always accompanies at least one manual sign (i.e., it does not appear by itself but may also not be left out), (ii) that the predicate generally falls under the scope of the headshake, and (iii) that headshake on the entire VP is possible. However, further possibilities for and constraints on spreading have not been explored in detail, and therefore, we will not gloss the headshake in the remainder of this article, leaving this issue, that is, the question in how far the headshake is grammaticalized in GESL, for future investigation.


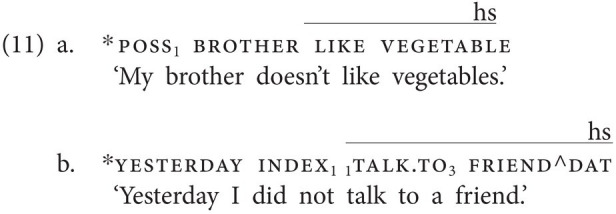


**Figure 2:**
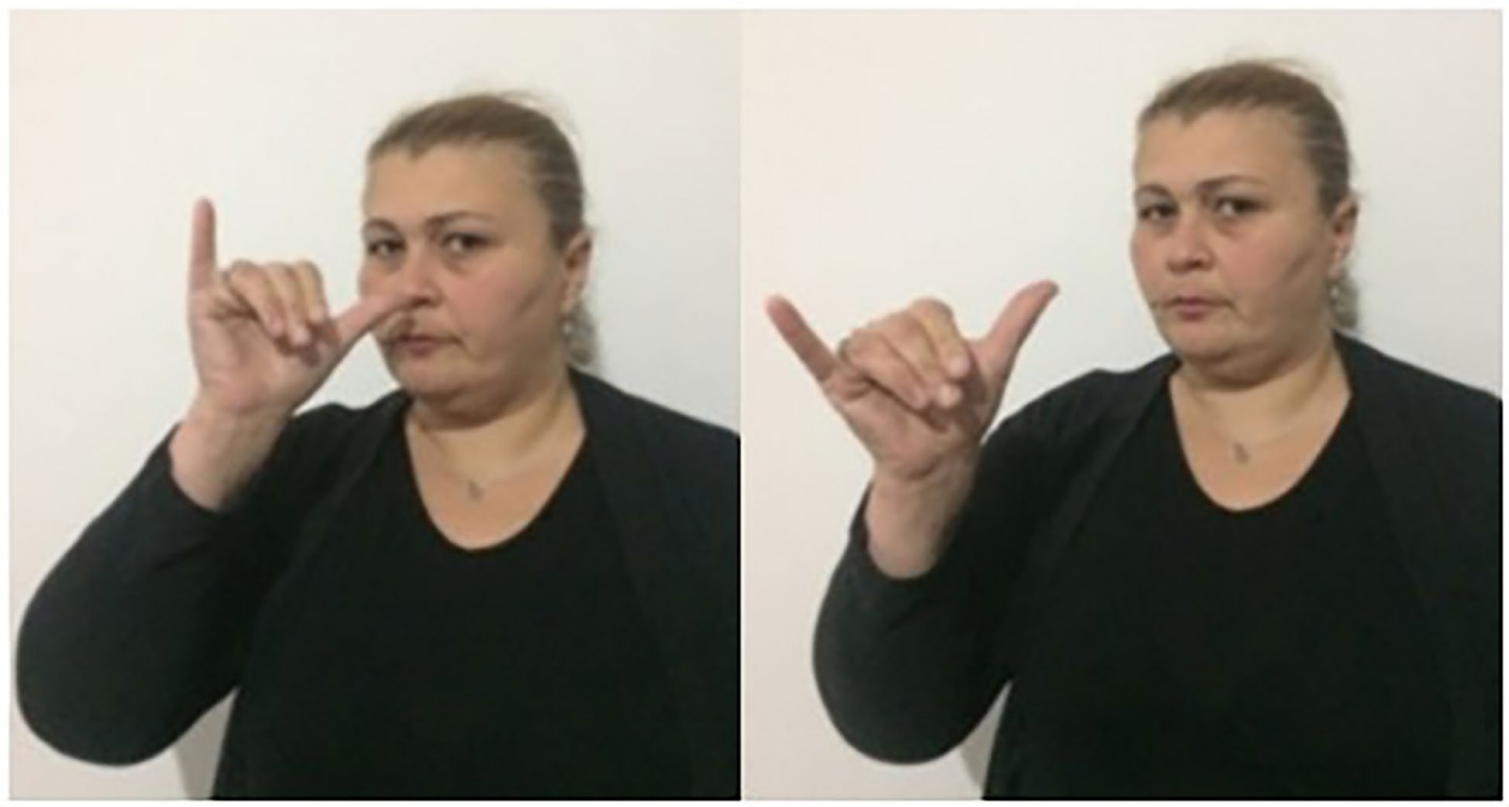
The negative particle neg-2 [“(can)not”].

GESL has a second negative particle which is widely used, and which behaves in exactly the same way as the particle *ver(a)* we described for Georgian [see (5c)]. That is, this particle, which we gloss as neg-2, has a modal flavor and can often be translated as “cannot” (deontically and epistemically); it is signed with a 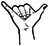-hand (thumb and pinky extended) which initially makes contact with the nose and moves forward, as illustrated in Figure 2. Crucially, this particle cannot combine with modal verbs (see “Negative Modals” for discussion), it always expresses the modal/circumstantial meaning by itself (Makharoblidze, 2019). The use of neg-2 is illustrated in (12). Similar to what we described for the clause negator neg-1, different word orders are possible; the particle may, for instance, follow (12a) or precede (12b) the verb.


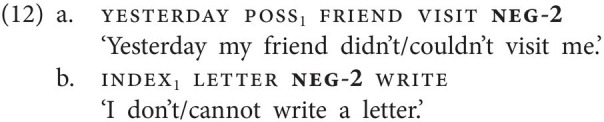


**Figure 3:**
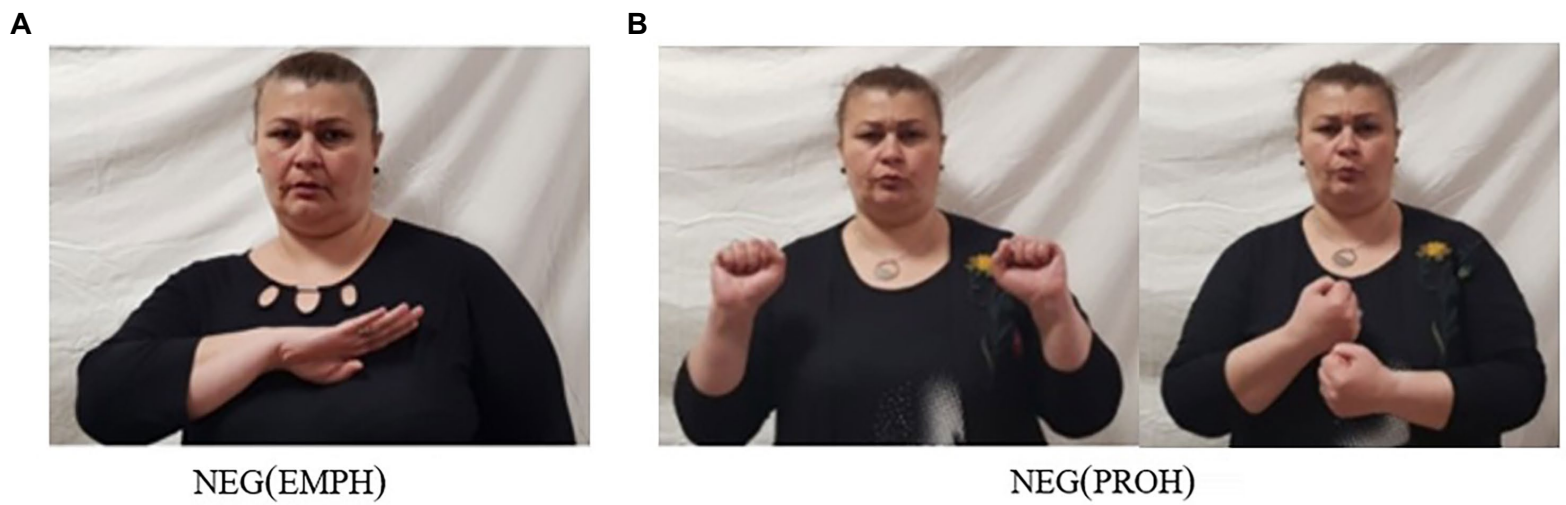
Two specialized negative particles **(A)** emphatic negative and **(B)** prohibitive marker.

Besides the two basic clause negators, GESL employs some specialized negative particles with additional semantics. One of these is the emphatic negator neg(emph), illustrated in Figure 3A. This particle, which appears to have grammaticalized from the two-handed sign dead, expresses strong negation (“really not”), as shown in (13a). The other one, which we gloss as neg(proh) and which is illustrated in Figure 3B, expresses a prohibitive meaning and is used in negative imperatives (13b). Both particles follow the verb.^5^


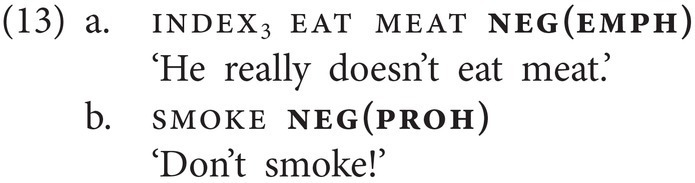


The usage of the particle neg(proh) resembles that of the particle *nu* that we described for Georgian in (8). It is thus possible that the existence of a dedicated prohibitive marker is the result of language contact. Remember, however, that while *nu* always precedes the verb, neg(proh) must follow the verb [but see (17a) below].^6^

### Negative Concord

Having established that GESL is a manual dominant sign language which features two basic negative particles and two negative particles with additional semantics, we now turn to Negative Concord. In GESL, just as in spoken Georgian, NC is attested, but not obligatory, in sentences involving neg-words like nothing or never. In (14), this is illustrated for both neg-1 and neg-2, occupying a postverbal position in an SOV structure (14a) or a preverbal position in an SVO structure (14b). We even came across examples in which three negative signs are combined (14c). In the remainder of this paper, we will not include patterns with three manual negative elements in our discussion of NC.


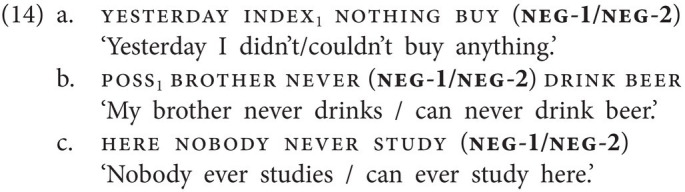


neg-1 and neg-2 can also combine within a clause, but only if neg-2 precedes neg-1 (15a–d). The resulting meaning is purely modal and can only mean “cannot.” Note further that there is only one postverbal slot for negation; hence a combination of postverbal neg-1 and neg-2 is ruled out, irrespective of order. The corresponding combination of particles, that is, of *ar(a)* and *ver(a)*, within a clause is not grammatical in spoken Georgian.


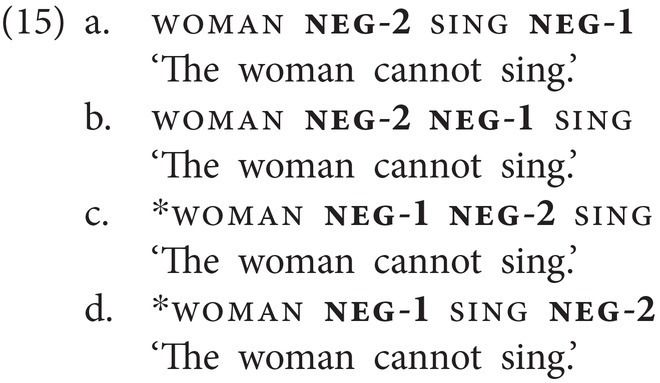


Furthermore, either of the two basic negative particles may combine with the emphatic negative particle neg(emph) within a clause, as shown in (16). In this case, the order of the particles is fixed in that neg-1/neg-2 must precede neg(emph).


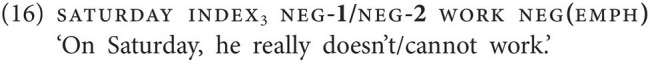


The prohibitive particle neg(proh) occasionally combines with the basic clause negator neg-1, yielding another type of NC. While neg(proh) always follows the verb when appearing by itself (13b), when combined with neg-1, it generally precedes the verb and neg-1 follows the verb (17a). However, in contrast to neg(emph), neg(proh) cannot co-occur with neg-2, as shown by the ungrammaticality of (17b). In Georgian, both corresponding combinations, i.e., of *nu* and *ar(a)* and of *nu* and *ver(a)*, would yield an ungrammatical sentence.


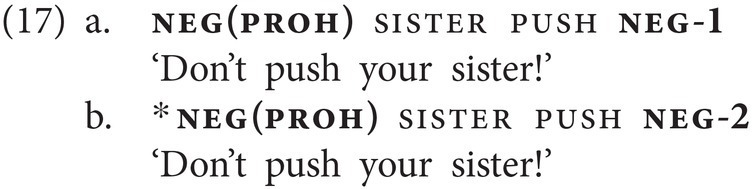


Note further (i) that neg(emph) and neg(proh) may not be combined within a clause, and (ii) that both these particles may combine with neg-words—similar to what we described for neg-1 and neg-2 (14). Actually, the combination of one of these four negative particles with a neg-word is the most commonly attested type of NC in GESL.

Examples that involve “doubling,” that is, the co-occurrence of two phonologically identical negators within a clause, would constitute another possible type of NC. In fact, this type has been reported for other sign languages, e.g., ASL (Petronio, 1993), Brazilian Sign Language (Libras; De Quadros, 1999), and Sign Language of the Netherlands (Van Boven et al., in press)—and at least for ASL and Libras, it has been argued to constitute a focus marking strategy. However, according to all our informants, NC of the doubling type is ruled out in GESL. In (18), this is illustrated for doubling of neg-1 and neg-2, but the ungrammaticality of doubling extends to other negative particles and neg-words.


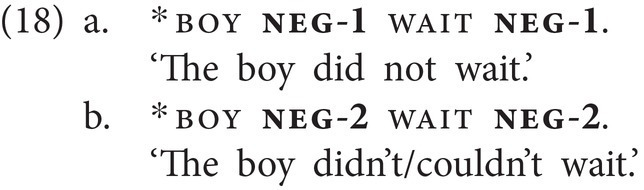


Taken together, we observe that GESL optionally allows for various types of NC, involving the basic negative particles (which may also combine with each other), neg-words, the emphatic negative particle, and the prohibitive particle. Yet, not all logically possible combinations are grammatical. We pointed out that NC is also optionally possible in Georgian. However, it is noteworthy that many of the combinations that are attested in GESL are ruled out in Georgian. Further types of NC will be addressed in “On the Interaction of Negation With Tense, Aspect, and Modality”, where we will also present an overview table of the attested combinations.

### Summary

Word order in GESL is rather free, and this freedom extends to the positioning of negative particles vis-à-vis the verb and object. While GESL shares the former property, flexible word order, with spoken Georgian, the latter property is clearly different from Georgian, where the negative particles must immediately precede the verb. The usage of a manual negative element is obligatory in GESL, that is, the language has to be classified as a manual dominant sign language. GESL has a rich inventory of negative particles. So far, we presented four particles, two of which, neg-1 and neg-2, we consider basic (although the latter comes with additional modal meaning), and two, neg(emph) and neg(proh), which carry additional meaning. Further particles will be introduced in the next section. Both GESL and Georgian optionally allow for Negative Concord but differ from each other with respect to which negative elements can be combined within a clause.

## On the Interaction of Negation With Tense, Aspect, and Modality

Having discussed the basic negation strategies of GESL, we now turn to a description of how negation interacts with other grammatical categories, *viz.* tense, aspect, and modality. The fact that negation commonly interacts with modal notions in interesting ways has been described for many spoken and signed languages (De Haan, 1997; Zeshan, 2004a; Iatridou and Zeijlstra, 2013; Homer, 2015, among others). In “Negative Modals”, we address dedicated negative modals that we identified in GESL. Subsequently, in Section “Tense- and Aspect-Specific Negative Particles”, we turn to the use of tense- and aspect-specific negative particles. Typological studies show that the usage of negators or negation strategies that are specific to certain tenses is not uncommon across spoken languages (e.g., Miestamo, 2005); however, to date, only few such cases have been described for sign languages. Finally, in “A Negation-Modality-Tense Interaction”, we address a typologically highly unusual three-way interaction between negation, modality, and tense, namely, a tense-specific occurrence of NC.

### Negative Modals

**Figure 4:**
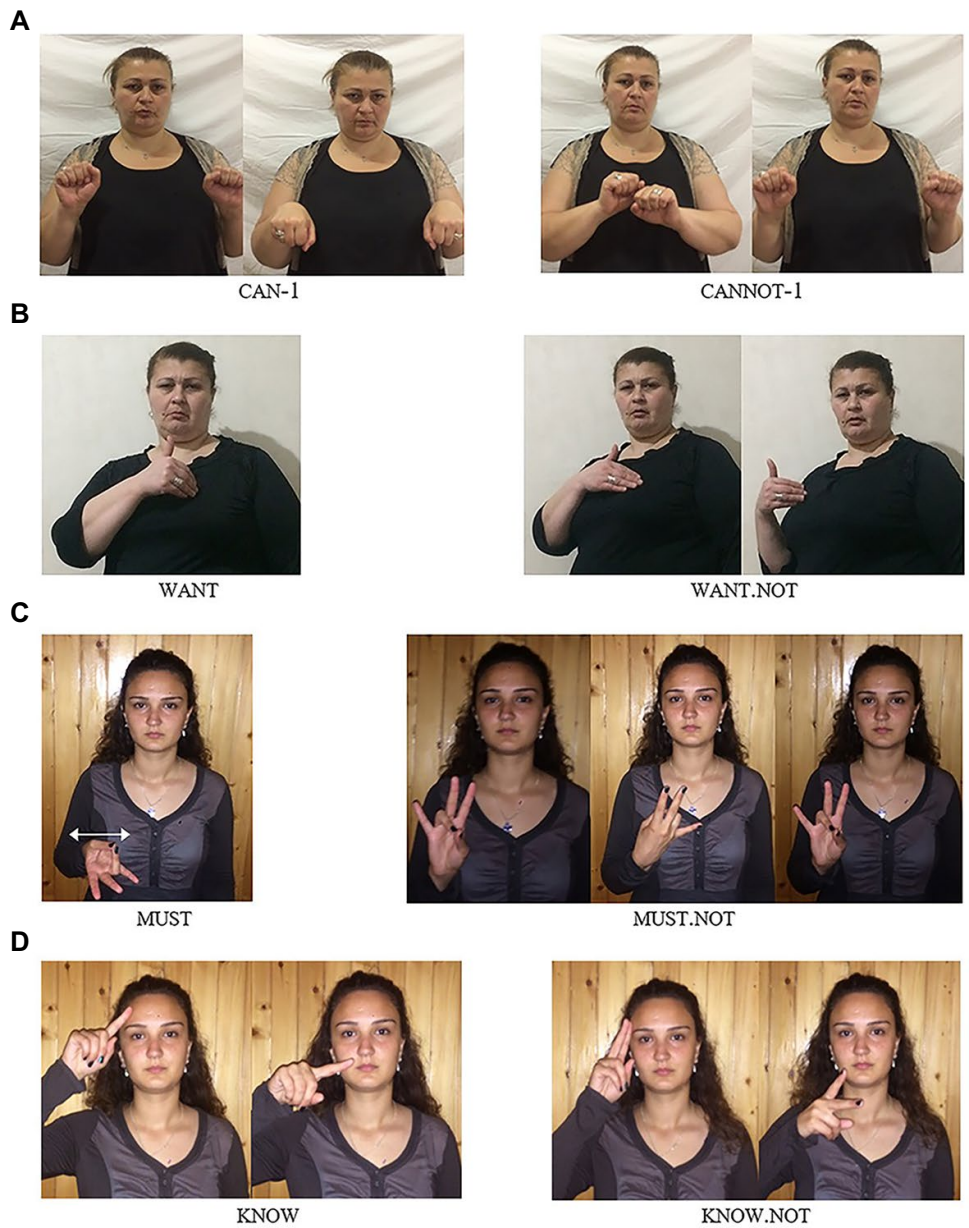
Modals and their negative counterparts in GESL: **(A)** can-1—cannot-1; **(B)** want—want.not; **(C)** must—must.not; and **(D)** know—know.not. (images in **A**, **B**, and **D** from Makharoblidze and Pfau, 2018, p. 141; © John Benjamins, reprinted with permission).

For many sign languages, it has been observed that they employ special forms of modal verbs in the context of negation (Shaffer, 2002; Zeshan, 2004a; Pfau and Quer, 2007). Such negative modals may result from cliticization of the basic clause negator to the modal, or they may be suppletive forms. GESL is no exception in this respect. Besides the basic negative particle neg-2, which, as pointed out above, may but does not have to introduce modal force, GESL has special negative forms for the modals can-1, want, must, and know.^7^ The four modals as well as their negative counterparts are illustrated in Figure 4.

The stills make clear that the formational changes observed in the negative forms differ from modal to modal: while cannot-1,^8^ want.not, and must.not are characterized by different types of movement changes, know.not involves a change in handshape. To be precise: can-1 involves a downward movement of two 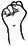-hands articulated at the wrist, while cannot-1 is articulated with a sideward movement of both hands; in want, the fingertips of the hand contact the contralateral side of the chest, while in want.not, a sideward movement to the ipsilateral side is added; in must, the palm of the hand (thumb contacts ring finger) is oriented upwards, and the sign involves a repeated sideward movement on the horizontal plane, while in must.not, the palm is initially oriented outward, and by rotating the lower arm, it is turned inward, then outward again; and finally, in know, the 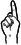-hand contacts the forehead and then moves downward, while in know.not, the 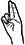-hand makes contact and changes into a 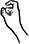-hand while performing the downward movement.

The forms in Figure 4 thus neither involve cliticization of one of the basic negators nor are they clear cases of suppletion, as most phonological aspects of the base signs are preserved [see Zeshan (2004a, p. 41–51) and Quer (2012, p. 320–323) for discussion of different types of “irregular negatives” across sign languages]. We therefore consider these as instances of partial suppletion which are characterized by simultaneous, i.e., stem-internal changes. In (19) and (20), we illustrate the use of the first two of these modals by means of glossed examples. Once again, the examples exemplify that different orders are attested. Note, however, that the SOModV order of (19) can also apply to the modal want/want.not and, vice versa, the SModVO order of (20) is also possible for can-1/cannot-1.


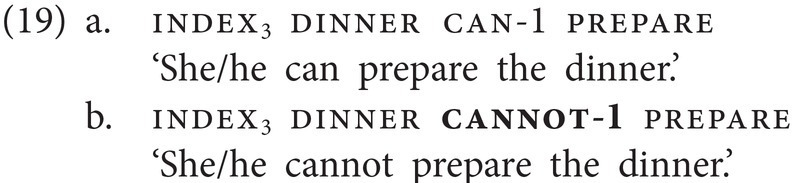



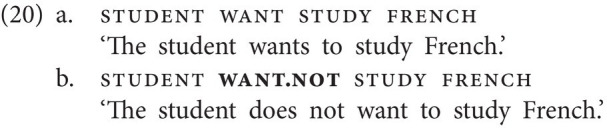


The examples in (21a,b) further reveal that NC involving a negative modal and one of the two basic clause negators is impossible. We only illustrate this for clause-final neg-1/neg-2, but the ungrammaticality is independent of the position of the negative particle. Crucially, however, we will demonstrate in “A Negation-Modality-Tense Interaction” that, quite strikingly, this ban on NC is lifted for neg-1 in past tense contexts. Furthermore, while the combinations illustrated in (21a,b) are ungrammatical, negative modals may combine with neg(emph), as shown for want.not in (21c).


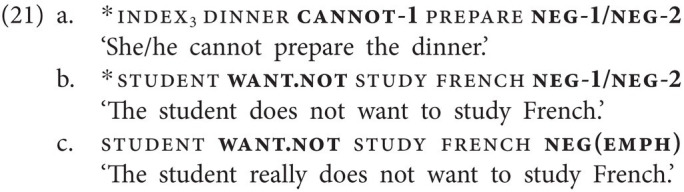


In clear contrast to GESL, Georgian does not employ specialized negative modals; rather modal verbs are negated in the same way as lexical verbs. In (22), we illustrate this only for the modal verb *dzl* (‘can’), but the same is true for other modal verbs. As is evident from (22b), the form of the modal remains the same; the only change observed is the addition of the negative particle. Note that modal verbs can only combine with the negative particle *ar(a)*, as the particle *ver(a)* itself is endowed with modal meaning.


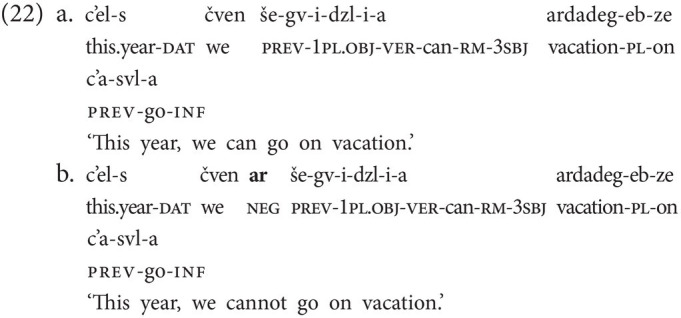


### Tense- and Aspect-Specific Negative Particles

**Figure 5:**
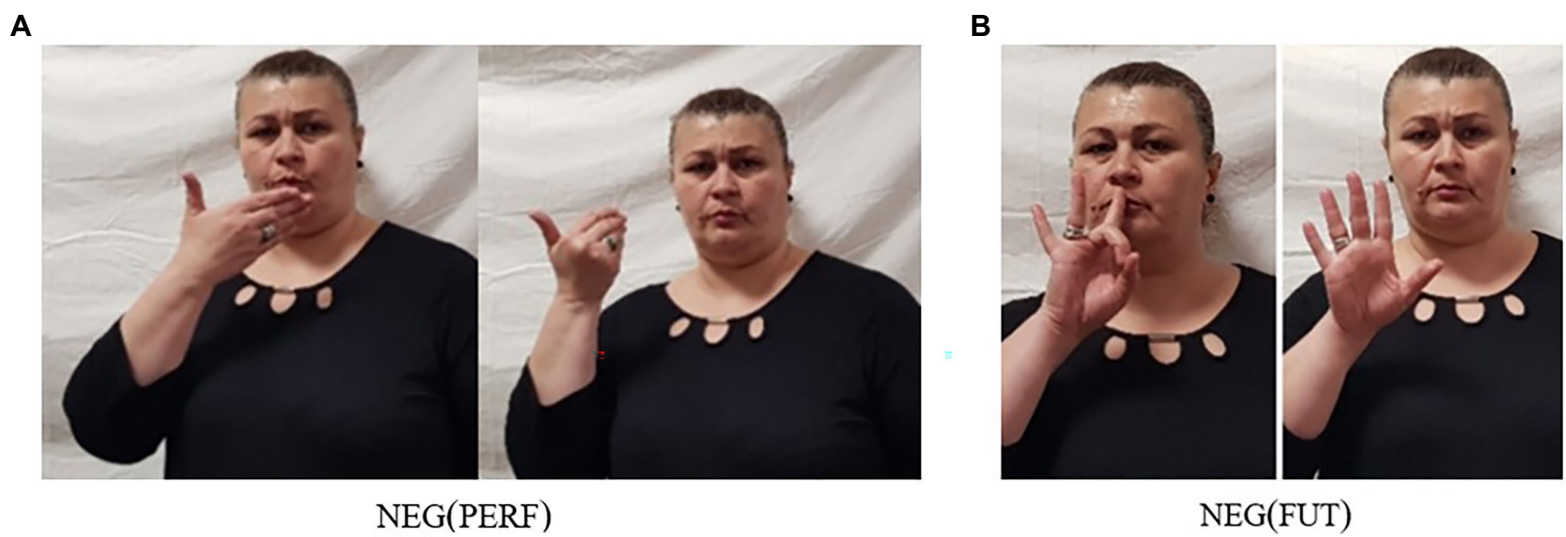
Tense- and aspect-specific negative particles in GESL: **(A)** neg(perf) and **(B)** neg(fut).

In the data we collected, we also encountered tense- and aspect-specific negative particles, another phenomenon that is not attested in spoken Georgian. The first of these particles is the particle neg(perf), illustrated in Figure 5A, which is clearly a mono-morphemic form and is used in perfective (or completive) contexts (23a). Crucially, the aspectual interpretation results from the use of the particle alone [*cf.* use of the particle not.yet in the NGT example in (3a)]. (23b) shows that, just like other negative particles, neg(perf) may also precede the verb, and that it may optionally combine with the basic clause negator neg-1 (note that the reverse order of the two particles would also be grammatical). However, in crucial contrast to the basic clause negator neg-1, neg(perf) cannot combine with neg-2 (23c).


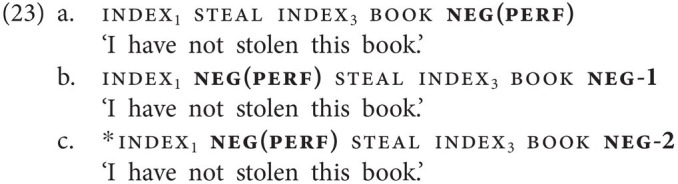


Next, to neg(perf), we came across the tense-specific particle neg(fut), which is only used in the future tense. Figure 5B illustrates that neg(fut) is a compound form by origin, involving the basic clause negator neg-1. However, the meaning of the first part is no longer transparent, and the second part has lost the side-to-side movement characteristic of neg-1. The sign only involves a short outward rotation of the hand during which the handshape changes. Use of this particle alone is sufficient to encode the temporal meaning and thus makes the use of the future tense marker future unnecessary (24a,b). Alternatively, the marker future can be used in combination with the basic negator neg-1 (24c), and also in combination with neg(fut), leading to double marking of future tense, as illustrated in (24d). Note further that, just like neg(perf), neg(fut) may also precede the verb and may combine with neg-1, but not with neg-2 (24e).^9^^,^^10^


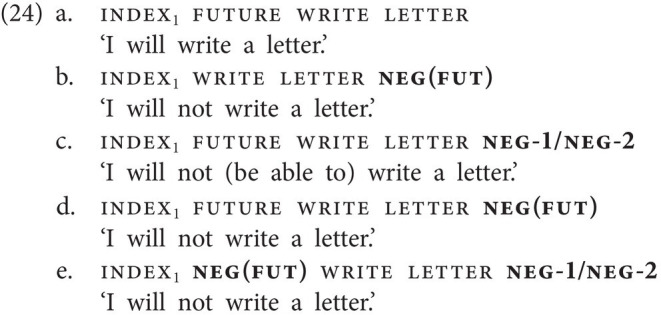


As already pointed out above, tense-specific negative particles (or negation strategies) are not uncommon in spoken languages. Makharoblidze and Pfau (2018, p. 147), for instance, observe that out of the 297 languages listed in the Appendix to Miestamo (2005), 53 (18%) display tense-specific negation strategies. Yet, when it comes to sign languages, the use of a tense-specific negative particle has to date only been reported for Israeli Sign Language (Meir, 2004). In contrast to the particle we described for GESL, the one identified for Israeli Sign Language carries a past tense meaning and is therefore glossed as neg-past. Yet, similar to what we described for GESL, Meir shows that use of neg-past alone yields the desired past tense reading (e.g., index_3_ sleep neg-past ‘He did not sleep at all’).

### A Negation-Modality-Tense Interaction

**Figure 6:**
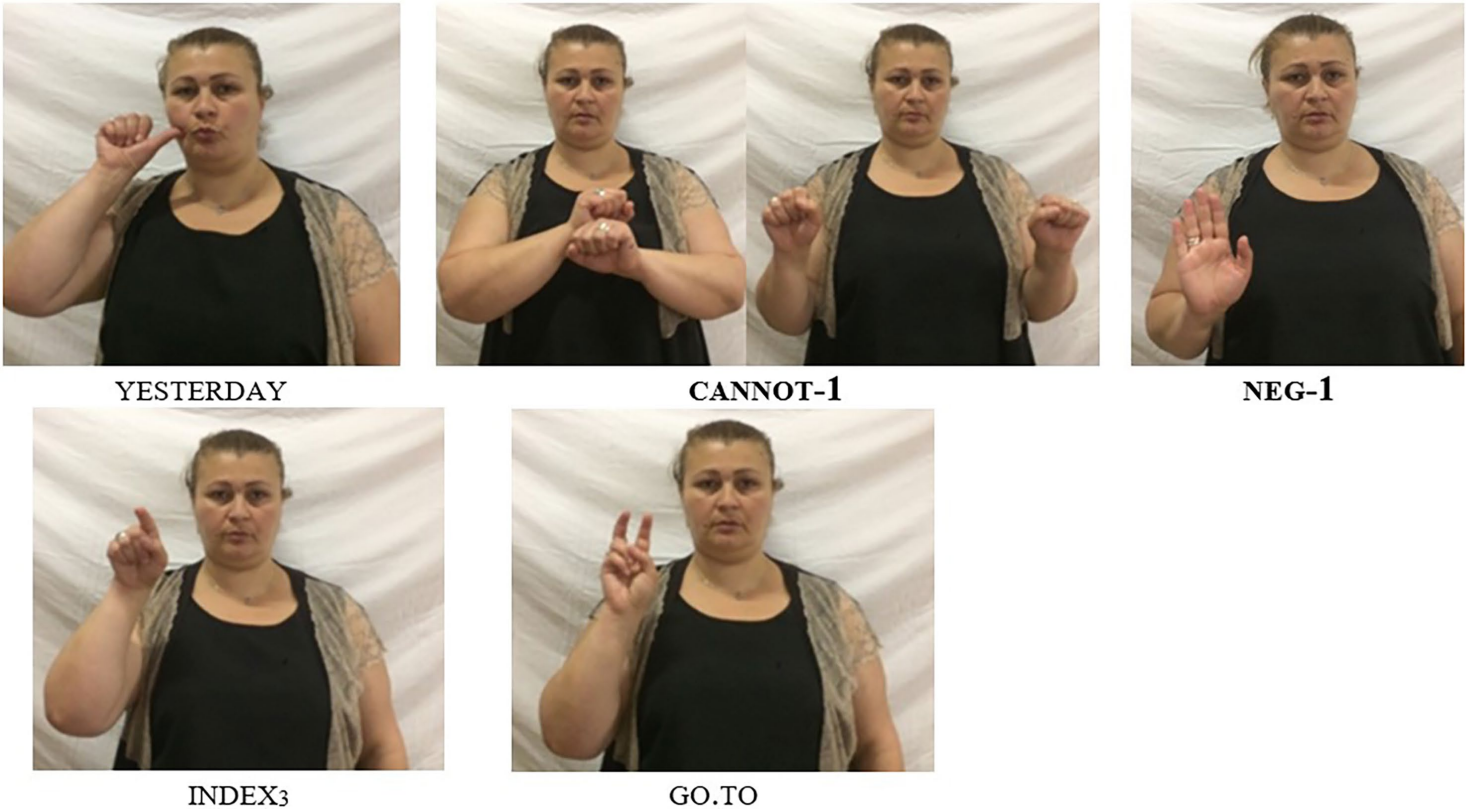
The negative modal cannot-1 used in a past tense context: ‘Yesterday it was impossible to go there/one could not go there’; note the combination of the irregular negative form cannot-1 with the negator neg-1 (slightly adapted from Makharoblidze and Pfau, 2018, p. 144; © John Benjamins, reprinted with permission).

**Figure 7:**
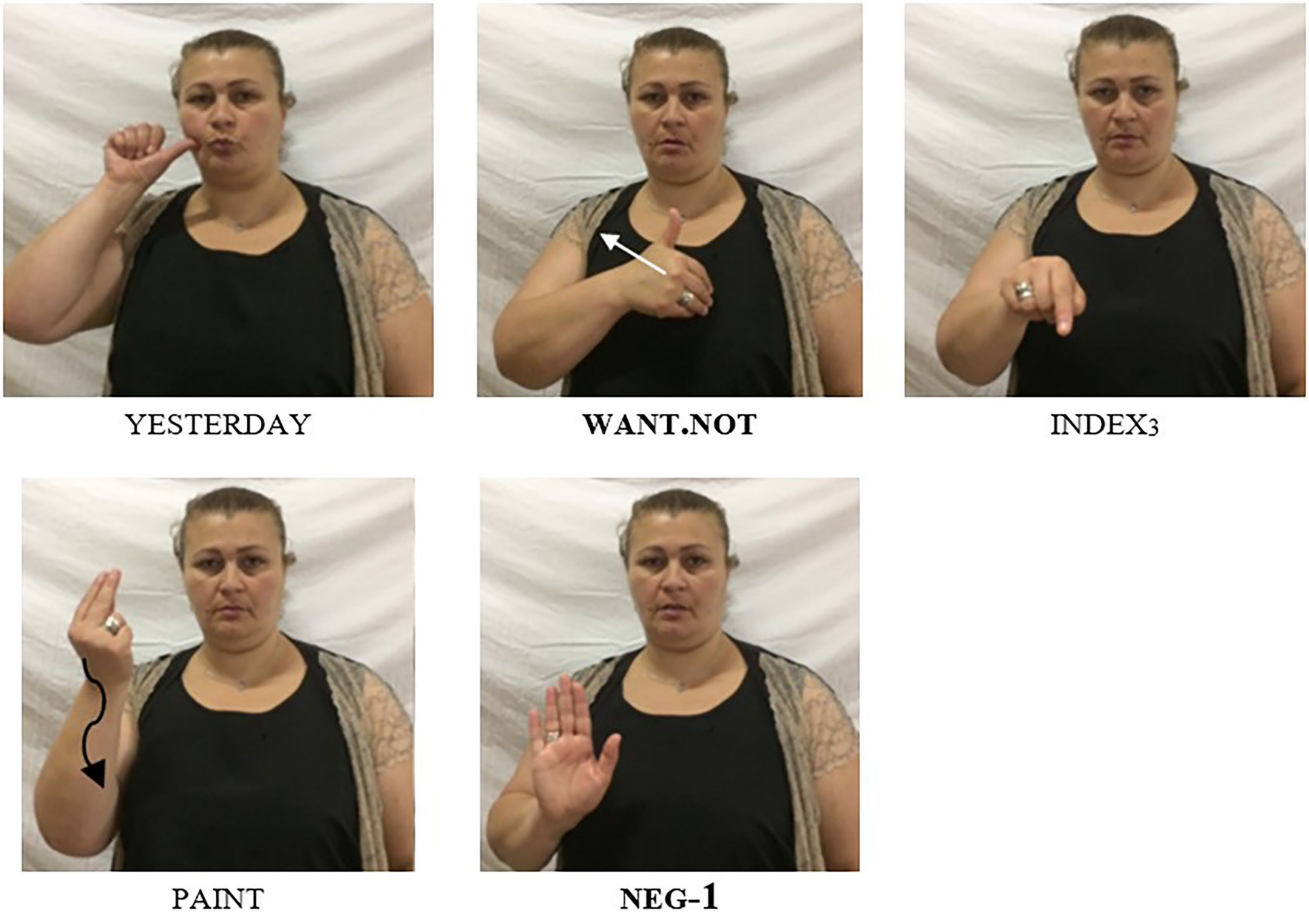
The negative modal want.not used in a past tense context: ‘Yesterday I did not want to paint it’; note the combination of the irregular negative form want.not with the negator neg-1 (slightly adapted from Makharoblidze and Pfau, 2018, p. 144; © John Benjamins, reprinted with permission).

In “Negative Modals”, we introduced negative modals, and we showed that these modals cannot combine with the basic clause negator neg-1. However, when studying GESL modal verbs in more detail and eliciting clauses with different tense specifications (as overtly indicated by adverbials) from native signers, Makharoblidze and Pfau (2018) noticed that in past tense contexts, the signers systematically combined the special negative form of the modal with the manual sign neg-1. In Figures 6, 7, we provide examples that illustrate this pattern for the negative modals cannot-1 and want.not, respectively. Once again, different orders are possible but the negative particle neg-1 must always follow the negative modal [similar to what we observed when it combines with neg-2; see (15)]. Figure 6 exemplifies the order (S)–neg.mod–neg-1–VP, while the order (S)–neg.mod–VP–neg-1 is illustrated in Figure 7.

The pattern we observe in Figures 6, 7 is in striking contrast to what we described for present tense examples in (21), where the combination of a negative modal and neg-1 leads to ungrammaticality. In (25a), we further illustrate this constraint with the present tense equivalent of the example in Figure 7 (we add an overt subject pronoun in order to make clear that the ungrammaticality does not result from the missing subject). It is thus evident that the ban on NC between a negative modal and neg-1 does not apply to all tenses.^11^ In fact, further discussions with the informants revealed that this type of NC is obligatory in past tense contexts, as shown by the ungrammaticality of (25b).


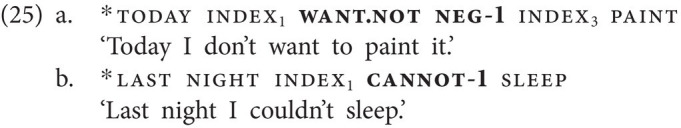


Makharoblidze and Pfau (2018) also offer a brief discussion of the GESL pattern from a cross-linguistic perspective. On the one hand, they show that NC involving negative modals has been described for some sign languages (e.g., ASL and NGT). Crucially, however, this type of NC is never constrained to a specific tense. On the other hand, they present examples from two spoken languages—Arapesh (a Torricelli language spoken in Papua New Guinea) and Lewo (an Austronesian language spoken on Vanuatu)—in which one tense is negated by a single marker, while another tense requires double marking. These examples, however, do not involve negative modals; rather, it is the basic negation strategy that differs dependent on tense.^12^ It thus appears that GESL presents us with a type of NC that has not previously been described for any signed or spoken language: obligatory, tense-specific NC involving negative modals.

### Summary

Beyond the basic and specialized (emphatic and prohibitive) negative particles discussed in “Basic Negation”, GESL also features two (maybe three) tense/aspect-specific negative particles as well as specialized negative modals, which we analyze as partially suppletive forms. Again, NC is attested, but it is severely constrained: both tense/aspect-specific particles may combine with neg-1 and neg(emph) but not with neg-2, and for obvious reasons, they cannot combine with each other; for semantic reasons, neg(proh) can only combine with neg(fut). Negative modals are particularly interesting in this respect, as they can combine neither with neg-1 nor with neg-2 in non-past contexts but must combine with neg-1 in the past tense. An overview of the combinatorial possibilities is provided in Table 1. Let us reiterate that almost all patterns reported in this section are clearly different from spoken Georgian, as Georgian neither features special forms for negative modals nor tense-specific negative particles.

**Table 1:** Possibilities for Negative Concord in Georgian Sign Language: “+” indicates that NC is attested; “–” indicates that NC involving these two elements is not attested.

	neg-1	neg-2	neg(emph)	neg(proh)	neg(perf)	neg(fut)	neg. modal	neg-word
neg-1	−	+^a^	+	+	+	+	−/+^b^	+
neg-2	+^a^	−	+	−	−	−	−	+
neg(emph)	+	+	−	−	+	+	+	+
neg(proh)	+	−	−	−	−	+	–^c^	+
neg(perf)	+	−	+	−	−	−	?^d^	+
neg(fut)	+	−	+	+	−	−	?^d^	+
neg. modal	−/+^b^	−	+	–^f^	?^d^	?^d^	–^e^	+
neg-word	+	+	+	+	+	+	+	−/+^f^

a neg-2 must precede neg-1.

b Only in past tense, but then obligatory.

c We have not attested any such examples, but this is arguably due to the fact that modals are in general unavailable in imperatives (and thus prohibitives).

d Further research is necessary, as different negative modals appear to behave differently when it comes to these combinations.

e The minus here refers to combinations of different negative modals as well as to cases of doubling, whereby the same negative modal appears twice in a clause.

f Different neg-words can be combined within a clause, but doubling of one and the same neg-word is ruled out.

Remember from the discussion above that doubling is ruled out in GESL [see (18)]—in Table 1, these are the cells that run diagonally from the top left to the bottom right. The only apparent exception are neg-words (bottom right cell), but crucially, the attested cases are not instances of doubling, as two different neg-words are involved [e.g., nobody and never in (14c)].

## Discussion

Now that we have given an overview of the rather complex and typologically unusual system of negation in GESL, we are going to investigate how this system compares to existing taxonomies of NC and double negation systems.

### Standard NC Systems in Spoken and Sign Languages

Generally speaking, languages vary cross-linguistically with respect to whether they allow NC or not. Dutch is a so-called Double Negation language, a language where every morpho-syntactically negatively marked element also induces a semantic negation. Consequently, in all three examples in (26), the co-occurrence of two neg-words yields an affirmative meaning.


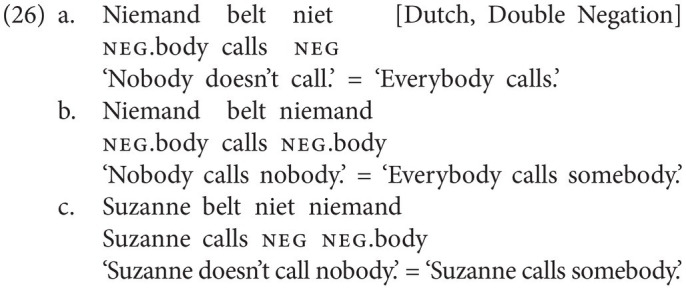


In contrast, Czech (27) and Italian (28) are NC languages, where one or more negative elements jointly yield one semantic negation. NC languages are commonly divided into so-called Strict NC languages and Non-strict NC languages (*cf.* Zeijlstra, 2004; Giannakidou, 2006). Czech is classified as a Strict NC language, as every neg-word—be it preverbal (i.e., VP-external) or postverbal (i.e., VP-internal)—obligatorily needs to be accompanied by the negative marker *ne*. In (27a), the neg-word appears in object position, while in (27b,c), it functions as subject and either precedes (27b) or follows (27c) the verb. Crucially, without the negative marker *ne*, all three sentences would be ungrammatical.


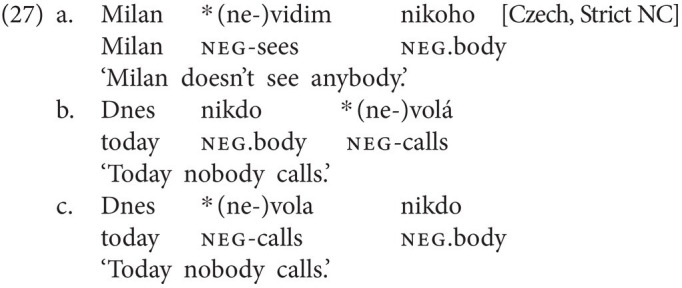


Italian, by contrast, is a so-called Non-strict NC language, as only postverbal (i.e., VP-internal) neg-words need to be accompanied by a higher negation, yielding an NC reading. Consequently, the examples in (28a) and (28c) pattern with the corresponding Czech examples in (27a) and (27c): both a neg-word in object position (28a) and a postverbal subject neg-word (27c) have to be accompanied by the negative marker *non*. However, in contrast to Czech, preverbal (i.e., VP-external) neg-words cannot be accompanied by a negative marker. Inclusion of a negative marker in examples like (28b) thus results in ungrammaticality (under neutral intonation).


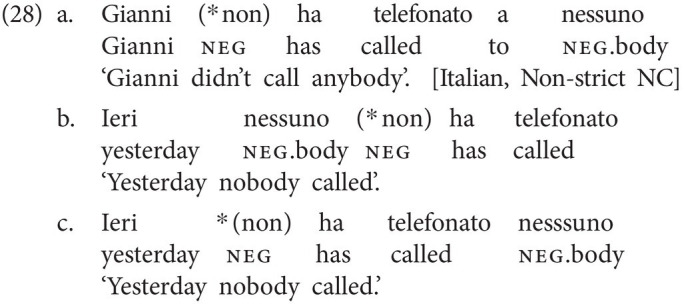


Strikingly, all three types of languages can be attested among sign languages as well, showing that the distribution of types of NC/DN languages is not specific to modality.

Like Dutch, LIS is a Double Negation language, where no (manual) negative element is accompanied by another one. Remember from the examples in (2) that LIS is a manual dominant sign language. According to Geraci (2006b), examples involving NC, consisting of a combination of the negative marker not^13^ and a neg-word, are straightforwardly ungrammatical, as shown in (29a,b). To the extent that a negative marker and a neg-word can co-occur in a clause, only a Double Negation reading is marginally available (29c; Geraci, 2006b; *cf.* also Pfau, 2016).^14^


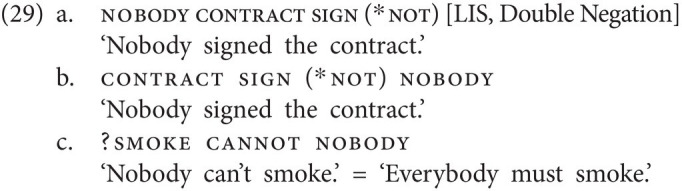


As was shown in (2), a non-manual headshake may accompany negation in LIS. Yet, given that a clause cannot be negated by means of the headshake only, the headshake, by definition, does not count as a negative marker and consequently cannot establish NC relations either.

Things are crucially different in (at least some) non-manual dominant sign languages, where neg-words inside and outside the VP (or more precisely, postverbal and preverbal neg-words) are accompanied by an additional negative marker, *viz.* the headshake. This is the case, for instance, in NGT, a non-manual dominant sign language, where the headshake can negate a clause by itself [see (1c)] and where, consequently, the combination of a neg-word and the headshake constitutes an instance of NC. As the examples in (30) illustrate, neg-words are indeed always accompanied by the headshake, regardless of whether they appear in pre- or postverbal position and regardless of whether they are subjects or objects.


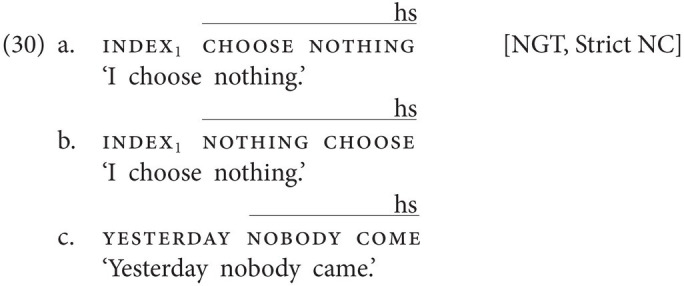


Russian Sign Language (RSL), finally, is a language where VP-external subject neg-words, which unlike in most spoken languages appear in a postverbal, sentence-final position, cannot be accompanied by a manual negative marker, but where VP-internal neg-words, subjects and objects alike, must be accompanied by the negative marker, just as is the case in spoken Non-strict NC languages (see Kuhn and Pasalskaya, in press; Kuhn, 2020).^15^ In (31), the VP-internal object neg-word nothing (31a) or the VP-internal subject neg-word nobody (31b) must be licensed by the sentence-final negative marker not, whereas a VP-external negative subject as in (31c) may not.


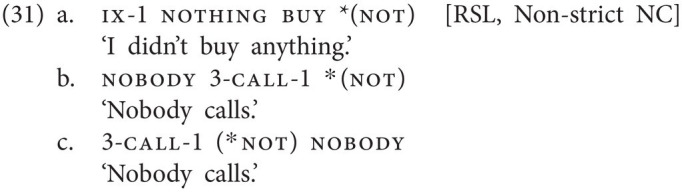


Hence, *prima facie*, the same dimensions of variation with respect to negation and NC that apply in spoken language also apply in sign languages, showing again that the latter only differ from the former in terms of their modality of symbolic realization.

### Non-standard NC Systems in Spoken and Sign Languages

In recent years, it has turned out, however, that the landscape of NC in spoken languages is much richer than sketched in the previous section. Without doing full justice to the literature, at least three other aspects of variation related to negation and NC are attested among spoken languages. These concern: (i) the optionality of NC; (ii) the co-occurrence of multiple negative markers; and (iii) hybrid NC systems, where only a strict subset of the set of negative elements can participate in NC relations. We discuss (i–iii) in turn.

First, in certain languages, NC is optional. West Flemish is a good example (*cf.* Haegeman, 1995; Haegeman and Lohndahl, 2010). Whereas neg-words may establish NC relations with both other neg-words (32a) or negative markers (32b) in this language, NC is never obligatory. Consequently, (32c) without NC is just as good as (32b).


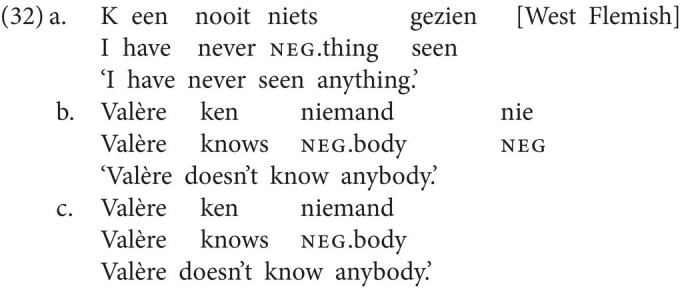


Second, albeit it is a rare phenomenon, in certain languages, neg-words must be accompanied by a negative marker but cannot establish an NC relation with each other. Whereas most spoken and signed NC languages, including Czech, Italian, and Russian Sign Language, exhibit NC constructions in which more than one neg-word participates, in Afrikaans, at least in its more conservative variety, every negative sentence, regardless of whether it contains a negative marker (33a) or a neg-word (33b), ends with the negative marker *nie* (*cf.* Den Besten, 1986; Biberauer, 2008, 2009; Biberauer and Zeijlstra, 2012). This means that Afrikaans allows not only NC between a neg-word and a negative marker (as in most other NC languages), but also between two negative markers.^16^


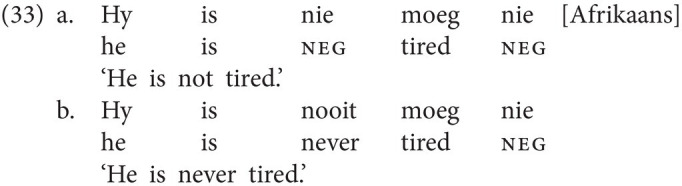


Third, in languages like French, as in most other NC languages, NC is possible between multiple neg-words, as shown in (34). However, French is exceptional in that any combination of neg-words with the negative marker *pas* gives rise to a Double Negation reading, irrespective of whether the neg-word appears in preverbal (35a) or postverbal position (35b). Note that the same holds for the combination of more than one neg-word with *pas*. In (35c), the two neg-words establish an NC relation to the exclusion of *pas*, and the sentence yields two semantic negations (see Zeijlstra, 2010):^17^


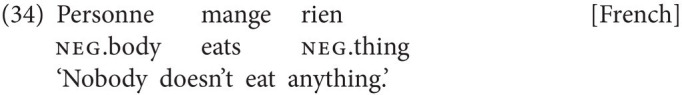



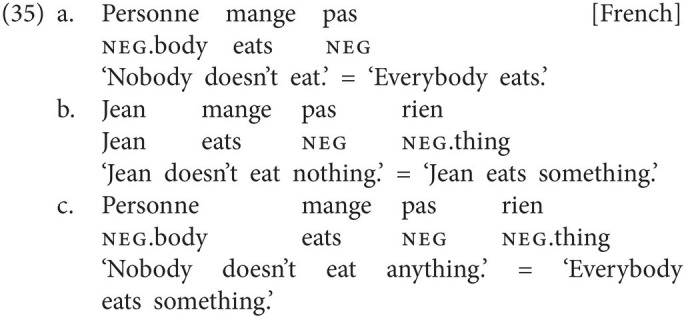


Irrespective of the exact underlying analysis, the examples above show that the landscape of NC is much richer than is generally assumed. This, of course, has strong repercussions for sign languages as well. If such atypical NC systems can be found in spoken languages, and there is nothing modality-specific about them, they should be expected to be manifest in sign languages as well. However, as of yet, such NC patterns have not been explicitly discussed in the literature.

Naturally, the question arises as to what constitutes the landscape of NC such that all the systems described above are possible. We would like to emphasize that, despite appearance, this landscape is not an “ordered mess” but follows from several constraints applying to the realization of negation in general. One such constraint is that negation should at least take structurally higher scope than the VP, and that not every negatively marked element, neg-words and negative markers alike, has to carry semantic negation. A full discussion of these facts is beyond the scope of this paper, but we refer to Zeijlstra (in press) for a detailed description of what are possible NC systems and what not. The crucial fact that is at stake here is that these constraints are not modality-specific and are therefore predicted to be in principle possible in sign languages as well. Strikingly, the above-described atypical instances of NC are indeed attested in GESL, thus confirming this prediction (see Van Boven et al., in press, for NGT).

### Toward a Classification of GESL

The discussion of GESL above shows that such non-standard NC properties are indeed attested in sign language. First, as shown in (14), repeated here as (36), NC is not obligatory in GESL, and the language thus patterns with West Flemish in this respect.


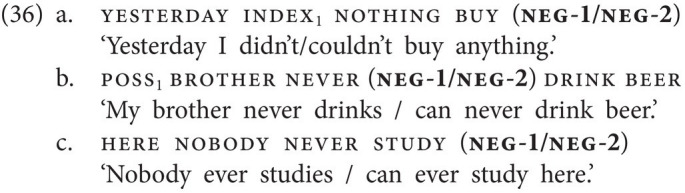


Second, as shown in (15a,b), repeated below as (37a,b), NC between two negative markers, here neg-1 and neg-2, is possible as well, yielding a pattern that is reminiscent of the one described for Afrikaans above.


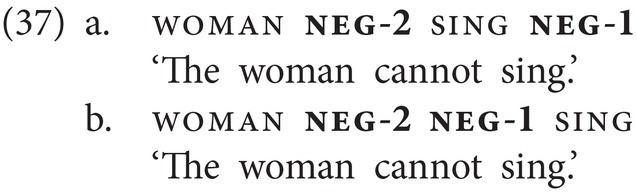


And, finally, as discussed at length in “On the Interaction of Negation With Tense, Aspect, and Modality”, and shown in Table 1, not every negative element may participate in NC relations. The examples in (21), repeated here as (38), for instance, show that negative modals, such as cannot-1 or want.not, cannot be accompanied by the negative markers neg-1 and neg-2.


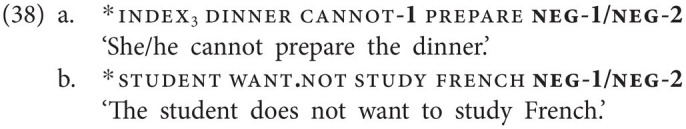


Hence, the outcomes of our investigation into a relatively unexplored sign language, GESL, show that the intricate and marked NC patterns observed in spoken languages like West Flemish, French, and Afrikaans can also be attested in sign languages.

Note finally, that the search for rare NC phenomena, which guided us from spoken languages to sign language, can, in principle, also go the opposite way. As discussed in “On the Interaction of Negation With Tense, Aspect, and Modality”, there is one context in GESL where NC is obligatory: when used in past tense contexts, negative modals have to combine with the negative marker neg-1, as is shown in (39; see also Figures 6, 7).


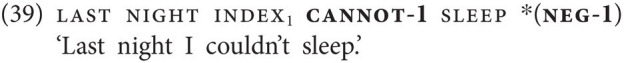


To the best of our knowledge, no such tense-governed instances of obligatory NC have hitherto been observed for spoken languages. Given the discussion above, it should come as no surprise that we take this current absence to be accidental and not to be a principled fact about sign language, spoken language, or linguistic negation in general.

## Conclusion

In this paper, we made a contribution to sign language typology, a young research field that pursues two, oftentimes related, goals (Pfau and Zeshan, 2016; Zeshan and Palfreyman, 2017). On the one hand, scholars strive to identify structural differences across sign languages, i.e., intra-modal differences, in all domains of grammar—think, for instance, of handshape inventories, patterns of pluralization, and relativization strategies (Perniss et al., 2007). On the other hand, some studies offer a cross-modal comparison, whereby the patterns that are identified are compared to patterns and classifications that have previously been established on the basis of typological research into spoken languages.

In our study on negation and Negative Concord in Georgian Sign Language, we pursued both these goals—following suit of previous studies which compared negation strategies across sign languages (e.g., Pfau and Quer, 2002; Zeshan, 2004a) and/or between sign and spoken languages (e.g., Pfau and Quer, 2002; Pfau, 2016; Gökgöz, 2021). As for the first goal, we established that GESL belongs to the class of manual dominant sign languages, which require the presence of a manual negator—a pattern that has been reported for various sign languages. What makes GESL typologically unusual, as compared to other sign languages, are (i) the availability of a rather wide variety of negative particles, including emphatic and tense-specific particles, and (ii) the multifarious, yet not unconstrained, possibilities for Negative Concord. As for the second goal, the comparison to spoken languages, we showed (i) that the attested negation patterns are clearly different from those available in spoken Georgian, that is, they are not the result of cross-modal borrowing, and (ii), zooming in on NC, that GESL displays some special and unusual characteristics of NC that have also been identified in several spoken languages. A typologically highly unusual characteristic of GESL—both in comparison with other sign languages and spoken languages—is the existence of a tense- and verb-specific type of NC, *viz.* obligatory NC with modal verbs in the past tense.

A component that we neglected in the present study is the non-manual marker involved in negation: a side-to-side headshake. The data allows us to ascertain that a headshake is commonly used in GESL negation and that it cannot by itself change the polarity of a clause. However, we are not yet in a position to say something about its scope, that is, whether it is capable of spreading beyond the manual negative sign. For a manual dominant sign language, the expectation would be that the non-manual marker is confined to the manual negator [*cf.* the LIS example in (2a)]. Yet, the available data suggest that in GESL, the headshake can extend over parts of the clause, e.g., the verb and/or the object. Further investigation of GESL might thus contribute to the typology of sign language negation, as it may reveal that there is also variation within the group of manual dominant sign languages—as has already been demonstrated for non-manual dominant sign languages (Pfau and Quer, 2002). The question would then be whether the headshake is a grammatical marker which is capable of spreading, as has recently been argued for Russian Sign Language (Rudnev and Kuznetsova, 2021), or whether its use is less constrained because it is a co-speech gesture rather than a grammatical element, as has been argued for Australian Sign Language by Johnston (2018).

## Data Availability Statement

The data analyzed in this study is subject to the following licenses/restrictions: While the participants gave consent for having screen shots from recordings published in the article (as well as for such screen shots being included in conference presentations), they did not consent to having the entire data set made publicly available. Requests to access these datasets should be directed to Tamar Makharoblidze: tamar.makharoblidze@iliauni.edu.ge.

## Ethics Statement

Ethical review and approval was not required for the study on human participants in accordance with the local legislation and institutional requirements. The patients/participants provided their written informed consent to participate in this study. Written informed consent was obtained from the individual(s) for the publication of any potentially identifiable images or data included in this article.

## Author Contributions

TM collected the GESL data by means of elicitation and grammaticality judgements. She wrote “Georgian Sign Language,” “Methodology” and Negation in Spoken Georgian,” which were revised based on feedback by RP. “Negative modals” and “A Negation-Modality-Tense Interaction” were co-authored by TM and RP. RP wrote “Sign Language Negation” and “Conclusion” as well as first versions of “Word Order and Basic Negation in GESL” and “Tense- and Aspect-Specific Negative Particles,” which were revised based on feedback by TM and HZ. HZ wrote “Discussion,” which was revised based on RP’s feedback. All authors contributed to the article and approved the submitted version.

## Funding

HZ’s contribution to this project was supported by a grant by the German Science Foundation (Deutsche Forschungsgemeinschaft—DFG), project number 414910159 (“Asymmetries and movement in spoken and sign languages”). Moreover, HZ received funding for publication fees through the DFG-funded Research Training Group (#2636: “Form-meaning mismatches”).

## Conflict of Interest

The authors declare that the research was conducted in the absence of any commercial or financial relationships that could be construed as a potential conflict of interest.

## Publisher’s Note

All claims expressed in this article are solely those of the authors and do not necessarily represent those of their affiliated organizations, or those of the publisher, the editors and the reviewers. Any product that may be evaluated in this article, or claim that may be made by its manufacturer, is not guaranteed or endorsed by the publisher.

## Footnotes

^1^We adopt common conventions for glossing sign language examples. Signs are glossed in small caps; the gloss index represents a pointing sign, poss a possessive pronoun; when two words are necessary to gloss a single sign, these are separated by a period (e.g., not.yet); the symbol “^” indicates cliticization; subscript numbers next to index or a verb sign represent loci in the signing space (1 = on or close to signer’s body and 3 = in neutral signing space); and lines above the gloss indicate the presence of a non-manual marker (in all our examples a headshake), the length of the line showing the scope of the marker.

^2^Other sign languages of the non-manual dominant type are, for instance, American Sign Language, Catalan Sign Language, Finnish Sign Language, and Indopakistani Sign Language, where a manual negative sign never renders a sentence negative by itself; the group of manual dominant sign languages includes, for instance, Hong Kong Sign Language and Jordanian Sign Language. Turkish Sign Language appears to present us with a hybrid type, as a manual negator is obligatory, but the relevant non-manual marker is capable of spreading (Gökgöz, 2011).

^3^An interesting observation that is not well investigated for Georgian and that we cannot go into here, concerns the fact that neg-words in object position prefer the preverbal position (6a), while in an NC structure, they normally follow the verb (6b).

^4^We are neglecting many important details here, which have triggered interesting discussions in the sign linguistics literature regarding the proper treatment of the spatial modification of verbs. For different theoretical accounts, see Padden (1988), Meir (2002), Liddell (2003), Lillo-Martin and Meier (2011), Pfau et al. (2018), and Schembri et al. (2018), among others.

^5^GESL has a lexical verb prohibit, which is not phonologically related to neg(proh) in any way. Note further that the negative modal must.neg (see Figure 4C) can also be used as a prohibitive marker.

^6^For the sake of completeness, let us add that GESL also features two negation strategies that appear to be derivational in nature. First, the sign empty can combine with nouns to yield a meaning comparable to the English negative suffix *-less* (e.g., heart^empty “heartless”). Second, the sign without can combine with signs of various lexical categories to express a meaning similar to the English prefix *un-* (e.g., work^without “unemployed”). More in-depth study is required, but it appears that both these signs have undergone grammaticalization. Note further that empty may also be used as a negative possessive, as in father house empty (‘Father does not have a house’), suggesting an intermediate stage on the grammaticalization path.

^7^know is a lexical verb in GESL, but—as in many other languages, including spoken Georgian—it is commonly understood and behaves like an epistemic modal: as we show here, it displays partial suppletion in the context of negation, and, as will be shown in “On the Interaction of Negation With Tense, Aspect, and Modality”, it also behaves like other modals in past tense contexts.

^8^As suggested by the gloss, there are alternative forms of the modal can (can-2 and can-3). These two forms are negated in a different way, i.e., by a combination of the previously introduced neg-2 with a flat hand. It is likely that this compound form, which occupies a postverbal position, results from a fusion of neg-2 with neg-1. We will not include can-2 and can-3 in the following discussion, but it is worth noting that different variants of the modal verb can may combine within a clause (e.g., girl can-1 dance can-2 ‘The girl can dance’).

^9^What we have to leave open for now is the combination of negative modals with either neg(perf) or neg(fut). Apparently, different modals behave differently in this respect; it seems, for instance, that neg(fut) can combine with want.not but not with cannot-1. For this reason, we include a “?” in the relevant cells in Table 1 in “Summary”.

^10^There is a third sign which might be analyzed as a tense-specific negative particle, namely, the sign which could be glossed as neg(pst). However, in contrast to the two signs in Figure 5, this is a transparent combination of two existing signs: the past tense copula was and the basic negator neg-1. We are therefore reluctant to analyze this sign, which in principle might also be glossed as was^neg-1, as a dedicated negative particle. Evidence that suggests that we might indeed be dealing with a more conventionalized form, possibly in the process of being grammaticalized, comes from the observation that the parts can never be separated; that is, a string like dress was beautiful neg-1 (implied meaning ‘The dress was not beautiful’) is ungrammatical, and the order would rather have to be dress beautiful was^neg-1. In other words: in such contexts, use of the conventionalized combination is obligatory. Further research is necessary to determine the exact present status of was^neg-1 / neg(pst).

^11^In contrast to the ban on NC between a negative modal and neg-2, which does apply to all tenses.

^12^Moreover, in the spoken languages, present and past tense are grouped together (realis) and distinguished from future (irrealis) in the context of negation, while in GESL, present and future tense align and contrast with past tense.

^13^LIS has two negative markers, which are glossed as non and neg by Geraci (2006b), and both of which appear in postverbal position. In the examples in (29), we subsume both markers under the gloss not. Geraci also notes that the two negative markers cannot co-occur in one clause.

^14^Geraci does not provide examples with not and neg-word in object position [i.e., examples that would correspond to (27a) and (28a)] but states that the ungrammaticality of (29a,b) extends to these cases [see Geraci (2006a) for relevant examples and discussion].

^15^Just like Italian Sign Language, Russian Sign Language is a manual dominant sign language when it comes to negation. A negative headshake may accompany a manual negative marker but cannot replace it. Such headshakes cannot render a sentence negative on their own and therefore are not real negative markers.

^16^The only exception to this generalization arises when two negative markers should appear adjacent to one another; in this case, only one *nie* is realized (see Biberauer (2008) for arguments that this scenario involves a real instance of haplology).

^17^French also has an optional preverbal negative marker *ne*, but as this element never renders a sentence negative by itself, it cannot count as an NC-item (or as a negative element in the first place), and we therefore leave it out from the examples.
